# Associations of Socio-Demographic Characteristics of Dairy Goat Farmers in Greece with Biosecurity-Related Practices in the Farms

**DOI:** 10.3390/ani14142136

**Published:** 2024-07-22

**Authors:** Daphne T. Lianou, George C. Fthenakis

**Affiliations:** Veterinary Faculty, University of Thessaly, 43100 Karditsa, Greece

**Keywords:** farm worker, health management, infection, quarantine, small ruminant, surveillance, transmission

## Abstract

**Simple Summary:**

This work studied the situation regarding biosecurity practices in goat farms in Greece and their potential associations with the socio-demographic characteristics of farmers. The findings indicated that, in goat farms, the socio-demographic characteristics of farmers are associated with the biosecurity level therein. This study identified that the presence of working staff on the farm and the length of farming experience were characteristics associated with the application of most biosecurity-related individual practices and procedures. Female farmers and the presence of working staff were associated with the overall level of biosecurity on the farms. This knowledge can be useful when developing biosecurity programs on goat farms.

**Abstract:**

Given the significance of goat farming in Greece, our study aimed to explore biosecurity conditions on goat farms and refers to an investigation performed on 119 farms countrywide in Greece. The objective of the present work was to evaluate and report on potential associations between the socio-demographic characteristics of goat farmers and practices that play a role in biosecurity on farms. Data were obtained during a large cross-sectional study performed across Greece in 119 goat herds. The socio-demographic characteristics of farmers, as well as 16 variables related to biosecurity, were recorded; these were practices or events that could potentially enhance or compromise biosecurity on farms. A score based on all the biosecurity-related variables was devised by assigning a ‘1’ or ‘−1’ mark for each variable recorded on a farm that could enhance or compromise, respectively, biosecurity. Among variables potentially enhancing biosecurity on farms, the application of disinfections on the farm and the maintenance of isolation for sick animals were practiced more frequently; among variables potentially compromising biosecurity on farms, the presence of hunters in the area around the farm and grazing practices for animals were recorded more frequently. The median overall score for biosecurity-related practices for all farms in the study was 1 (interquartile range: 2.5; minimum: −4; maximum: 5). Among socio-demographic characteristics, the presence of working staff on a farm was associated with five individual biosecurity-related practices and the length of farming experience with three individual biosecurity-related practices. In the multivariable analysis, female farmers (*p* = 0.007) and the presence of working staff on the farm (*p* = 0.025) emerged as the two significant socio-demographic characteristics with an association with the overall biosecurity level on farms. This knowledge can be useful when developing biosecurity programs on goat farms. Recognition of locally applied farm-level practices enhancing biosecurity could form a basis for farmers to apply more rigorous and effective relevant plans.

## 1. Introduction

The World Health Organization has described biosecurity as the accumulation of strategic and integrated approaches for the management of relevant risks to human, animal, and plant health [1]. Hence, the maintenance and development of biosecurity policies, procedures, and practices aim ‘*to enhance the ability to protect human health, agricultural production systems, and the people and industries that depend on them*’ [1]. In the context of animal health and production, biosecurity refers to a set of precautions with the purpose of protecting livestock, mainly from pathogens but also from invasive species [2,3,4,5]. Biosecurity measures and practices can be relevant at country, region, farm, or individual levels [4,6,7]. Biosecurity interventions are also applied in order to prevent the transmission of pathogens from livestock to people [8].

Goat farms are particularly prone to the transmission of pathogens, as for most of them, grazing is an integral part of their general management system. During grazing, a variety of pathogens can be transmitted to animals during direct contact with goats of other herds in the area; among these, *Mycoplasma ovipneumoniae* is transmitted most frequently through direct contact between animals [9] (‘nose-to-nose’ [10]), whilst *M. mucoides* and *M. capricolum* are transmitted mainly via aerosol droplets [9,11]. Pathogens can also be transmitted through ingestion during the grazing of goats on pasture, for example, the larvae of gastrointestinal nematodes (e.g., *Teladorsagia circumcincta* or *Haemonchus contortus* [12]). Finally, pathogens can also be contracted by animals during grazing itself; a notable example is *Dichelobacter nodosus*, the causal pathogen of footrot [13].

A recent (June 2024) literature evaluation performed by the authors using the topic search string [[*biosecurity*] AND [goat* OR *caprine* OR *Capra hircus*]] revealed 113 relevant items. A detailed individual evaluation of these items revealed a total of 60 articles that covered various facets of the topic. Among these, only three have reported farmers’ perspectives and attitudes on occurrence, control, and communication related to biosecurity procedures and approaches. These were carried out in Sweden [14], Kenya [15], and Australia [16] and reported the significance of farmers’ experience and relevant education as determinants for the standards of biosecurity applied on goat farms. It is notable that despite the importance of the goat industry for the agricultural sector in Greece, which has the largest goat population and is the largest goat milk producer in Europe [17], the topic of biosecurity on goat farms has not been studied. This lack of studies related to biosecurity on goat farms may reflect the nature of goat farming in the country; notably, most goat farms in Greece operate under a semi-extensive or extensive management system [18], which can hinder the establishment and application of specific biosecurity measures.

Given the significance of goat farming in Greece, our study aimed to explore the biosecurity conditions on goat farms and refers to an extensive investigation performed on 119 farms countrywide in Greece. The objective of the present work was to evaluate and report on potential associations between the socio-demographic characteristics of goat farmers and practices that play a role in biosecurity on farms.

## 2. Materials and Methods

### 2.1. Visits to Goat Farms and Interviews of Farmers

**Figure 1:**
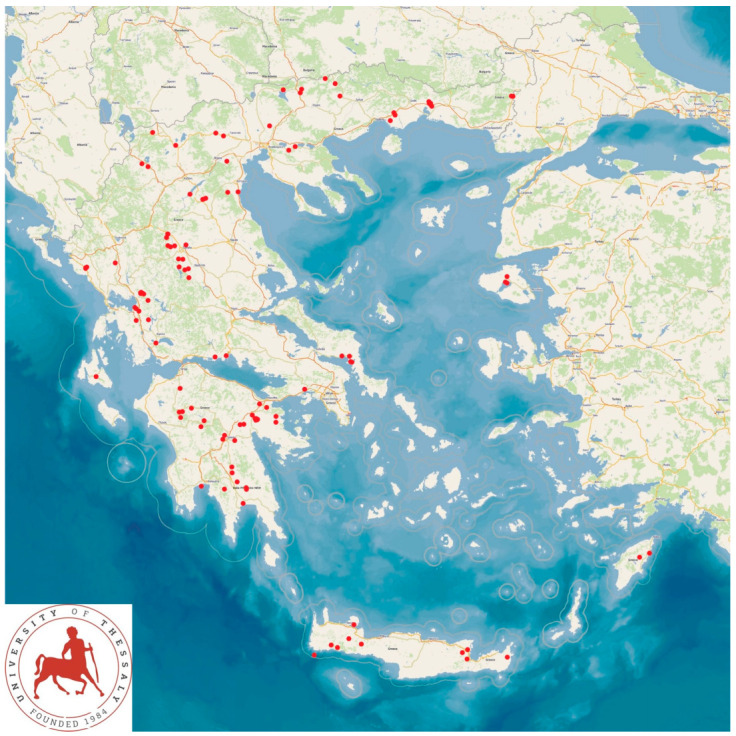
Map presenting locations of the 119 goat farms throughout Greece which were visited during the cross-sectional study.

The data were obtained during a large cross-sectional study performed across Greece (April 2019–June 2020) and in all 13 regional units of the country (Figure 1). The study involved 119 goat herds. The protocols for farm inclusion in the study have been presented in detail before [19].

No kind of relationship had existed between the researchers and any of the farmers prior to the visit. The researchers visited all 119 farms that participated in order to collect all relevant information. At the start, the objectives and the details of the study were presented to the farmers and discussed with them. Then, an interview was carried out by means of a structured, detailed questionnaire; the interview was always carried out by the same researcher (author D.T.L.) [19]. If farmers requested clarifications on the questions during the interview, these were provided immediately.

### 2.2. Data Management and Analysis

The following socio-demographic characteristics were recorded during the interview: farmer gender, age and education level, length of farming experience, professional involvement in farming, daily period of presence on the farm, family farming tradition, and presence of working staff on the farm (Table S1). Further, a total of 16 variables related to biosecurity were additionally recorded during the interview. These were practices or events that could potentially enhance (*n* = 9) or compromise (*n* = 7) biosecurity on farms (Table S1). The general characteristics of farms (e.g., management system applied, no. of animals) were also recorded (Table S1).

An overall score based on all the above biosecurity-related variables was devised by assigning a ‘1’ or ‘−1’ mark for each variable recorded on a farm that could enhance or compromise, respectively, biosecurity. Thus, the overall score ranged from −7 to 9 on each farm.

Data were entered into Microsoft Excel^®^ (Microsoft Corporation, Redmond, WA, USA) and analyzed using SPSS^®^ v. 21 (IBM Analytics, Armonk, NY, USA). Initially, descriptive analyses were carried out.

All continuous data used in the statistical analyses were assessed by means of the Shapiro–Wilk test. The results of testing showed that the distribution of these data had a significant departure from normality: *W* ≤ 0.98 (*p* > 0.075 for all assessments).

Outcomes in the form ‘*Occurrence of xxx*’ were considered (where xxx: each of the biosecurity-related variables (Table S1)). Initially, the significance of predictors among the socio-demographic characteristics of farmers was assessed in a univariable analysis by using cross-tabulation with Pearson’s chi-square test or Fisher exact test (for categorical variables) or the Mann–Whitney test (for continuous variables) with simple logistic regression. Then, a multivariable model was developed for each of the above outcomes, and parameters (i.e., the socio-demographic characteristics of farmers) found with *p* < 0.2 in the preceding univariable analyses were used in this model. The number of socio-demographic characteristics included in the models developed varied from 0 (variables: availability of footbath, availability of ditch at the main entrance, carcass disposal according to relevant regulations, application of rodenticides) to 6 (variable: grazing practice). Then, the socio-demographic characteristics were progressively removed from the model by using backwards elimination. A likelihood ratio test was performed to assess the *p*-value of each socio-demographic characteristic; among those found with *p* > 0.2, the one with the largest *p* was removed from the model. This procedure was repeated until no variable with *p* > 0.2 could be removed from the model. The socio-demographic characteristics included in the final assessment in each multivariable model are presented in Table S2. Their number varied from 0 (variables: availability of footbath, availability of ditch at the main entrance, carcass disposal according to relevant regulations, application of rodenticides) to 3 (variables: availability of fencing, application of systemic disinfections, grazing practice, presence of hunters in the area around the farm).

Subsequently, the outcome ‘*Overall score for biosecurity-related variables*’ was considered. Initially, the significance of predictors among the socio-demographic characteristics of farmers was assessed in a univariable analysis by using Spearman’s rank correlation. Then, a multivariable model was developed. Three socio-demographic characteristics were included in that model: farmer gender, length of farming experience, and presence of working staff on the farm. The same procedure described above was followed. The two variables included in the final assessment in the multivariable model were farmer gender and presence of the working staff on the farm (Table S2).

In all analyses, statistical significance was defined at *p* < 0.05.

## 3. Results

### 3.1. Descriptive Findings

Among the variables potentially enhancing biosecurity on farms, the application of disinfections on the farm and the maintenance of isolation for sick animals were the ones practiced most frequently. Among the variables potentially compromising biosecurity on farms, the presence of hunters in the area around the farm and grazing practices for animals were recorded most frequently. Details are given in Table 1.

**Figure 2:**
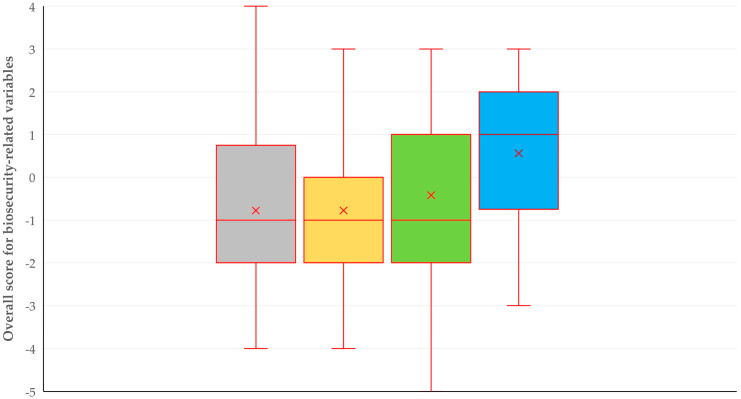
Box plots of the overall score for biosecurity-related practices on 119 farms in Greece, in accordance with the location of the farms (gray: farms in North Greece; yellow: farms in Central Greece; green: farms in South Greece; blue: farms on the islands).

The median overall score for biosecurity-related practices for all farms in the study was −1 (interquartile range: 3; minimum: −5; maximum: 4). There was a trend of higher scores on farms on islands (*p* = 0.055) (Figure 2); no other associations were found between the overall score for biosecurity-related practices and the characteristics of the farms (*p* ≥ 0.19) (Table 2).

### 3.2. Associations of Socio-Demographic Characteristics with Biosecurity-Related Practices

**Figure 3:**
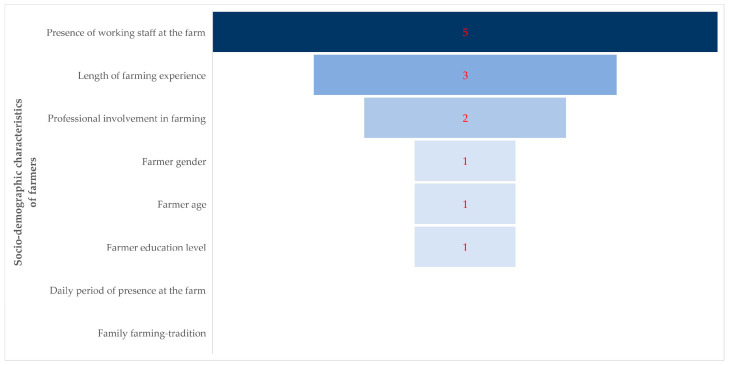
Funnel-type graph with the number of biosecurity-related practices with which significant associations were found with the socio-demographic characteristics of farmers.

Details of the univariable analyses for the associations of the various socio-demographic characteristics with biosecurity-related practices are given in Tables S3–S18. The results of the subsequent multivariable analyses for each outcome are summarized in Table 3. Among the various socio-demographic characteristics, the presence of working staff on the farm was associated with five biosecurity-related practices and the length of farming experience with three (*p* = 0.06 for differences between the various characteristics) (Figure 3).

### 3.3. Association of Socio-Demographic Characteristics with Overall Score for Biosecurity-Related Practices

**Figure 4:**
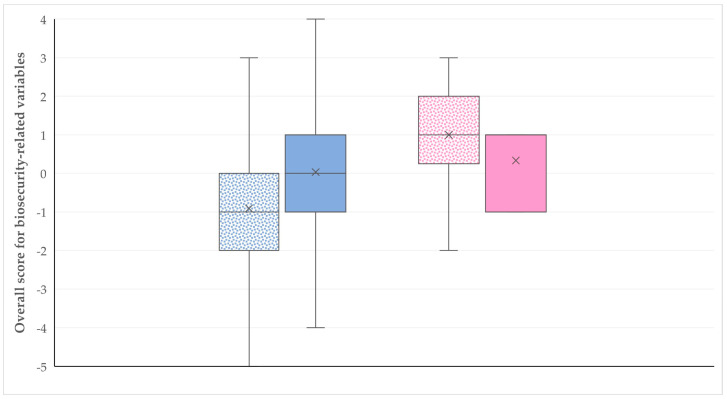
Box plots of the overall score for biosecurity-related practices on 119 farms in Greece in accordance with farmer gender (blue: male; pink: female) and the presence of working staff therein (no presence of working staff: motif pattern; presence of working staff: full pattern).

Details of the univariable analyses for the associations of the various socio-demographic characteristics with the overall score for biosecurity-related practices are given in Table S19. In the multivariable analysis, female gender (*p* = 0.007) and the presence of working staff on the farm emerged as significant variables among the socio-demographic characteristics of farmers (*p* = 0.022) (Figure 4, Table 4).

## 4. Discussion

This study focuses on the associations of the socio-demographic characteristics of farmers with biosecurity practices in goat farms, which may potentially affect the overall biosecurity level in a goat herd. This study also contributes to understanding the demographic characteristics of farmers’ engagement with animal health-related biosecurity practices that should be applied on farms.

Community (i.e., social) trust can be an important component in attempts within a country to set up systems for disease prevention, as these would require a communal approach to tackling the problem [21]. However, social trust in Greece has been found to be low, with the country being ranked 17th among 18 European countries [22,23]. This may influence the willingness of local farmers to take into account and align with regulations and common rules, to collaborate with veterinary authorities, and to trust others in achieving a common goal, in this case, disease prevention, through the implementation of policies and procedures for the benefit of all. This is reflected in the fact that many farmers declared that they did not follow biosecurity rules established through legal provisions, for example, the disposal of carcasses of dead goats.

In this study, we devised and employed a binary scoring system (yes/no) for the various biosecurity-related practices taken into account. In this context, practices enhancing biosecurity were scored positively (+1) if practiced, whilst practices with negative implications for biosecurity were given a negative score (−1) if practiced. That way, within each farm, the overall level of biosecurity applied therein was scaled.

The higher biosecurity scores on farms located on the islands of the country reflect the lack of wildlife mammals on them. This contributes to minimizing threats that originate from wild mammals. Such threats include the transmission of various pathogens, for example, rabies virus from foxes and wolves or *Chlamydia* spp. from wild cervids [24,25]. Goats are particularly vulnerable to attacks by predators, as they often browse individually among bushes and scrubs, which can facilitate attacks by predators [26], and indeed, the work of Petridou et al. [27] found that the diet of wolves often includes goats.

With regard to the identification of staff as a significant determinant for the application of a higher number of individual biosecurity-related practices on the farms, this can be associated with the fact that various practices aiming to decrease disease risks require relevant manual effort and time, for example, the regular application of disinfections or the maintenance of quarantining pens in farms. Goat farms in Greece are of the dairy production type, which adds a milking routine to the daily tasks to be carried out. Hence, a lack of working staff on the farm would lead to a low prioritization of tasks related to biosecurity. This finding underlines the idea that protection against disease risk and the enhancement of biosecurity measures require increased resources at various levels.

Improved biosecurity practices by female farmers were previously reported by Backhans et al. [28] on pig farms. Indeed, on dairy farms, women farmers declared a higher frequency of intention to abide by and implement biosecurity practices [29], which may explain the current findings. In this context, it is also notable that similar findings have been reported in studies related to human health [30,31].

Moreover, the association of the length of farming experience with the level of biosecurity practices has been reported among sheep farmers in the United Kingdom [32] and among pig farmers in Europe, including Belgium, Poland, the United Kingdom, Finland [33] and Sweden [28], as well as among horticultural farmers in Australia [34]. This is interesting given the variations in animal species farmed, management systems applied, and diseases occurring in the various countries, as well as in the differences in the legislation applied between countries.

Moreover, it is notable that the higher education and training level of farmers has also been associated with better compliance with procedures and rules associated with biosecurity, according to Racicot et al. [35], possibly because of better understanding by such farmers of, for example, the transmission of microorganisms or the consequences of impaired biosecurity procedures. Within this context, farmers with a better understanding of the idea of biosecurity may feel more confident in its benefits and apply better relevant practices [36]. Hence, in addition to increased staff at farms, we also wish to indicate the importance of developing training and training material for farmers and farm workers on the topic of farm biosecurity.

The context of biosecurity is extensive [7] and can include further practices than those evaluated in the present study. Indeed, the lack of standardized biosecurity protocols for goat farms, like those devised and implemented in the pig and poultry industries, makes the formulation of concrete and precise guidelines for goat farms difficult. This reflects the lack of scientific knowledge specifically pertaining to and applicable to goat farms.

Overall, the findings of this study underline the significance of human resources in maintaining high biosecurity standards in goat herds. Potentially, veterinarians, veterinary authorities, and veterinary associations and societies could be involved in improving biosecurity standards on farms. That way, different aspects of biosecurity practices could be communicated effectively among stakeholders.

## 5. Conclusions

The findings indicate that, on goat farms, the socio-demographic characteristics of farmers are associated with the biosecurity level therein. This knowledge could be useful when developing biosecurity programs on goat farms. Recognition of locally applied farm-level practices enhancing biosecurity could form a basis for farmers to apply more rigorous and effective relevant plans.

## Figures and Tables

**Table 1:** Descriptive findings regarding biosecurity-related variables recorded on 119 goat farms in Greece.

Variables	Goat Farms (*n* = 119) ^1^
Processing of farm waste	0.8% (0.2–4.6%)
Availability of fencing	45.4% (36.7–54.3%)
Type of fencing available (among farms with availability of fencing)	Light wire fence: 51.9%. Strong wire fence: 48.1% ^2^
Availability of footbath	8.4% (4.6–14.8%)
Availability of ditch at the main entrance	0.0% (0.0–3.1%)
Maintenance of quarantine period for new animals on the farm	51.3% (42.4–60.1%)
Maintenance of isolation for sick animals	82.4% (74.5–88.2%)
Carcass disposal according to relevant regulations	42.0% (33.5–51.0%)
Type of carcass disposal employed (among farms with carcass disposal resources according to relevant regulations)	Incineration: 18.0%. Safe burial: 78.0%. Both practices: 4.0% ^2^
Application of rodenticides	42.0% (33.5–51.0%)
Application of systemic disinfections	85.7% (78.3–90.9%)
No. of systemic disinfections performed annually (among farms which applied systemic disinfections)	2 (2) occasions ^3^
Grazing practice	94.1% (88.4–97.1%)
Duration of grazing annually (among farms with grazing practice)	12 (6) months ^3^
Common grazing of farm animals with wildlife ruminants	9.2% (5.2–15.8%)
Presence of wild carnivore mammals ^4^ near the farm	82.4% (74.5–88.2%)
Presence of hunters in the area around the farm	95.0% (89.4–97.7%)
Distance from the farm (among farms with presence of hunters in the area around them)	0.5 (1.9) km ^3^
Transhumance	23.5% (16.8–31.9%)
Presence of spots suitable for vector reproduction and development ^5^	69.8% (61.0–77.3%)
Purchase of replacement animals	34.5% (26.5–43.4%)

^1^ proportion of farm (95% confidence intervals) areas or median (interquartile range) (according to type of data); ^2^ proportion of farms among those that applied the practice; ^3^ median (interquartile range) with regard only to farms that applied the practice; ^4^ bears, foxes, jackals, and wolves; ^5^ muddy surfaces within or near the farm premises or adjacent to water troughs, manure buildups, or trenches with farm waste or manure.

**Table 2:** Overall score for biosecurity-related variables recorded on 119 goat farms in Greece, in accordance with the characteristics of the farms.

Characteristics of Farms	Overall Score	*p*
Farms applying intensive management ^2^	1 (3.0) ^1^	0.23
Farms applying semi-intensive management	0 (1.0)	
Farms applying semi-extensive management	−1 (3.0)	
Farms applying extensive management	−1 (2.0)	
Farms in North Greece	−1 (2.3)	0.055
Farms in Central Greece	−1 (2.0)	
Farms in South Greece	−1 (2.5)	
Farms on the islands	1 (2.3)	
Altitude of location of farm	*r_sp_* = −0.120	0.19
Number of animals on farm	*r_sp_* = 0.042	0.65
Years since establishment of farm	*r_sp_* = −0.092	0.32
Collaboration with veterinary practice	0 (1.8)	0.70
No collaboration with veterinary practice	−1 (3.0)	

^1^ median (interquartile range); ^2^ management types applied to farms, with description given according to the European Food Safety Authority classification [20].

**Table 3:** Summary of results of multivariable analyses for associations of socio-demographic characteristics of farmers with biosecurity-related practices on 119 goat farms in Greece.

Biosecurity-Related Practices	Socio-Demographic Characteristics of Farmers	*p*
Processing of farm waste	-	>0.08
Availability of fencing	-	0.07
Availability of footbath	-	-
Availability of ditch at the main entrance	-	-
Maintenance of quarantine period for new animals on the farm	Length of farming experience	0.0003
	Presence of working staff on the farm	0.001
Maintenance of isolation for sick animals	Presence of working staff on the farm	0.008
Carcass disposal according to relevant regulations	-	-
Application of rodenticides	-	-
Application of systemic disinfections	Presence of working staff on the farm	0.026
Grazing practice	Presence of working staff on the farm	0.004
	Length of farming experience	0.006
Common grazing of farm animals with wildlife ruminants	Farmer by profession	0.047
Presence of wild carnivore mammals near the farm	Gender	0.0007
Presence of hunters in the area around the farm	Farmer age	0.006
	Farmer education level	0.017
	Farmer by profession	0.023
Transhumance	-	>0.06
Presence of spots suitable for vector reproduction and development	-	>0.10
Purchase of replacement animals	Presence of working staff on the farm	0.0003
	Length of farming experience	0.003

**Table 4:** Results of multivariable analysis for socio-demographic characteristics with significant association with the overall score for biosecurity-related practices on 119 goat farms in Greece.

Variables	Odds Risk (±se) ^1^	*p*
Farmer gender		0.007
Female (1 (2.0)) ^2^	reference	-
Male (−1 (2.3))	4.293 ± 1.727	0.009
Presence of working staff on the farm		0.022
Yes (0 (2.0))	reference	-
No (−1 (2.0))	2.200 ± 1.424	0.028

^1^ se: standard error; ^2^ median (interquartile range) of overall score for biosecurity-related practices.

## Data Availability

Detailed results associated with this study are presented in the Supplementary Materials.

## Supplementary Materials

The following supporting information can be downloaded at https://www.mdpi.com/article/10.3390/ani14142136/s1: Table S1: List of variables related to biosecurity, the socio-demographic characteristics of farmers, and other characteristics recorded on 119 goat farms in Greece; Table S2: Details of multivariable models employed for the evaluation of potential associations between biosecurity-related practices and the socio-demographic characteristics of farmers on 119 goat farms in Greece; Table S3: Association between the processing of farm waste on 119 goat farms in Greece and the socio-demographic characteristics of the farmers; Table S4: Association of the availability of fencing on 119 goat farms in Greece with the socio-demographic characteristics of the farmers; Table S5: Association of the availability of a footbath on 119 goat farms in Greece with the socio-demographic characteristics of the farmers; Table S6: Association of the availability of a ditch at the main entrance on 119 goat farms in Greece with socio-demographic characteristics of the farmers; Table S7: Association of the maintenance of a quarantine period for new animals on the farm on 119 goat farms in Greece with the socio-demographic characteristics of the farmers; Table S8: Association of the maintenance of isolation for sick animals on 119 goat farms in Greece with the socio-demographic characteristics of the farmers; Table S9: Association of carcass disposal according to relevant regulations on 119 goat farms in Greece with the socio-demographic characteristics of the farmers; Table S10: Association of the application of rodenticides on 119 goat farms in Greece with the socio-demographic characteristics of the farmers; Table S11: Association of the application of systemic disinfections on 119 goat farms in Greece with the socio-demographic characteristics of the farmers; Table S12: Association of grazing practices on 119 goat farms in Greece with the socio-demographic characteristics of the farmers; Table S13: Association of common grazing of farm animals with wildlife ruminants on 119 goat farms in Greece with the socio-demographic characteristics of the farmers; Table S14: Association of the presence of wild carnivore mammals near 119 goat farms in Greece with the socio-demographic characteristics of the farmers; Table S15: Association of the presence of hunters in the area around the farm on 119 goat farms in Greece with the socio-demographic characteristics of the farmers; Table S16: Association of transhumance on 119 goat farms in Greece with the socio-demographic characteristics of the farmers; Table S17: Association of the presence of spots suitable for vector reproduction and development on 119 goat farms in Greece with the socio-demographic characteristics of the farmers; Table S18: Association of the purchase of replacement animals on 119 goat farms in Greece with the socio-demographic characteristics of the farmers; Table S19: Association of the overall score for biosecurity-related practices on 119 goat farms in Greece with the socio-demographic characteristics of the farmers.
