# Supplementary material for: Associations of Socio-Demographic Characteristics of Dairy Goat Farmers in Greece with Biosecurity-Related Practices in the Farms
Source: Animals (Basel). 2024 Jul 22;14(14):2136. doi: 10.3390/ani14142136 (PMC11273592; doi:10.3390/ani14142136)
Supplement: Supplementary file 1 [file animals-14-02136-s001.zip › animals-3088737-supplementary.pdf]

Associations of Socio-Demographic Characteristics of Dairy Goat Farmers in Greece with Biosecurity-Related Practices in the Farms

Dafni T. Lianou and George C. Fthenakis

Table S1. List of variables related to biosecurity, of socio-demographic characteristics of farmers, and of other characteristics recorded in 119 goat farms in Greece.

Variables related to biosecurity in farms
Processing of farm waste (yes / no)
Availability of fencing at the farm (yes / no)
Type of fencing at the farm (light wire fence / strong wire fence / stoned wall / wooden wall / other)
Availability of footbath (yes / no)
Availability of a ditch at the main entrance (yes / no)
Availability of disinfectant at the main entrance ditch (yes / no)
Maintenance of quarantine period for new animals into the farm (yes / no)
Maintenance of isolation for sick animals (yes / no)
Type of carcass disposal for dead animals (incineration / burying / feeding to dogs / feeding to birds / drop-off away)
Application of rodenticides (yes / no)
Application of systemic disinfections in the farm (yes / no)
Annual frequency of systemic disinfections in the farm (no. of occasions)
Grazing practiced (yes / no)
Duration of grazing during (i) the winter (no. of months) and (ii) the summer (no. of months)
Visual contacts of the farmer with wildlife mammals (yes / no)
Wildlife mammals identified within a radius of 2 km of the farm (description)
Common grazing of goats with wildlife mammals (yes / no)
Species of wildlife mammals identified in common grazing (description)
Presence of hunters in the area around the farm (within a radius of 2 km of the farm) (yes / no)
Distance from the farm that hunting activity occurs (km)
Transhumance (seasonal transfer of animals to other site) (yes / no)
Presence of spots suitable for vector reproduction and development (yes / no)
Types of spots identified (muddy spots inside or outside the buildings, near the water troughs / spots of wet manure / ditches with manure)
Source of replacement animals (own animals / purchase)

Socio-demographic characteristics of farmers
Gender (male / female)
Age (years)
Length of previous animal farming experience (years)
Highest general education level achieved (primary / secondary / tertiary)
Farmer by profession (yes / no)
Daily period of presence at the farm (hours)
Family tradition in farming (yes / no)
Presence of working staff at the farm (yes / no)
Other characteristics of farms
Production type (dairy / meat / wool / reproduction / other)
Management system (EFSA classification: shepherding / intensive / semi-intensive / semi-extensive / extensive / very extensive / mixed)
Year of the initial establishment of the farm (year)
No. of ewes (no.)
No. of rams (no.)
Collaboration with a veterinarian (yes / no)

Table S2. Details of multivariable models (*n* = 16) employed for the evaluation of potential associations of biosecurity-related practices with socio-demographic characteristics of farmers in 119 goat farms in Greece.

Outcome	Variables		
	**assessed in uni-variable analyses (*n*)**	**offered to the multi-variable models (*n*)**	**required in the final models**
Processing of farm waste	8	2	(a) Farmer age, (b) Presence of working staff at the farm
Availability of fencing	8	4	(a) Farmer by profession, (b) Farmer education level, (c) Presence of working staff at the farm
Availability of footbath	8	0	-
Availability of ditch at the main entrance	8	0	-
Maintenance of quarantine period for new animals into the farm	8	4	(a) Length of previous animal farming experience, (b) Presence of working staff at the farm
Maintenance of isolation for sick animals	8	1	(a) Presence of working staff at the farm
Carcass disposal according to relevant regulations	8	0	-
Application of rodenticides	8	0	-
Application of systemic disinfections	8	3	(a) Farmer by profession, (b) Farmer education level, (c) Presence of working staff at the farm
Grazing practice	8	6	(a) Length of previous animal farming experience, (b) Daily period of presence at the farm, (c) Presence of working staff at the farm
Common grazing of farm animals with wildlife ruminants	8	2	(a) Farmer by profession, (b) Daily period of presence at the farm
Presence of wild carnivore animals near the farm	8	3	(a) Farmer age, (b) Farmer by profession
Presence of hunters in the area around the farm	8	4	(a) Farmer age, (b) Farmer by profession, (c) Farmer education level
Transhumance	8	5	(a) Daily period of presence at the farm, (b) Farming family tradition
Presence of spots suitable for vector' reproduction and development	8	1	(a) Farmer by profession

Purchase of replacement animals	8	4	(a) Farmer experience, (b) Presence of working staff at the farm
Overall score for biosecurity-related variables’	8	3	(a) Farmer gender, (b) Presence of working staff at the farm

Table S3. Association of processing of farm waste in 119 goat farms in Greece with socio-demographic characteristics of the farmers.

**Farms with no processing of farm waste (*n* = 118)**			**Farms with processing of farm waste (*n* = 1)**			***p***
Male	Female		Male	Female		
107	11		1	0		0.75
Median age			Median age			
46.5 (17.0) years			26.0 (0.0) years			0.10
Mean farming experience			Mean farming experience			
25.0 (15.0) years			0.0 (0.0) years			0.17
Full-time	Part-time		Full-time	Part-time		
104	14		1	0		0.71
Daily time spent at the farm			Daily time spent at the farm			
15.0 (5.0) hours			8.0 (0.0) hours			0.24
Primary education	Secondary and vocational education	Tertiary education	Primary education	Secondary and vocational education	Tertiary education	
20	88	10	0	1	0	0.84
Farming family tradition	No farming family tradition		Farming family tradition	No farming family tradition		
103	15		1	0		0.70
Farm worker employment	No farm worker employment		Farm worker employment	No farm worker employment		
33	85		1	0		0.11

Table S4. Association of availability of fencing in 119 goat farms in Greece with socio-demographic characteristics of the farmers.

**Farms with no fencing (n = 65)**			**Farms with fencing (n = 54)**			p
Male	Female		Male	Female		
60	5		48	6		0.52
Median age			Median age			
43.0 (19.0) years			48.0 (15.0) years			0.09
Mean farming experience			Mean farming experience			
25.0 (15.0) years			30.0 (21.2) years			0.40
Full-time	Part-time		Full-time	Part-time		
54	11		51	3		0.06
Daily time spent at the farm			Daily time spent at the farm			
15.0 (5.0) hours			15.0 (5.0) hours			0.99
Primary education	Secondary and vocational education	Tertiary education	Primary education	Secondary and vocational education	Tertiary education	
7	53	5	13	36	5	0.13
Farming family tradition	No farming family tradition		Farming family tradition	No farming family tradition		
57	8		47	7		0.91
Farm worker employment	No farm worker employment		Farm worker employment	No farm worker employment		
15	50		19	35		0.15

Table S5. Association of availability of footbath in 119 goat farms in Greece with socio-demographic characteristics of the farmers.

<table>
<tr><th colspan="6">Farms with no footbath (n = 109)</th><th colspan="6">Farms with footbath (n = 10)</th><th>p</th></tr>
<tr><td colspan="3">Male</td><td colspan="3">Female</td><td colspan="3">Male</td><td colspan="3">Female</td><td></td></tr>
<tr><td colspan="3">100</td><td colspan="3">9</td><td colspan="3">8</td><td colspan="3">2</td><td>0.22</td></tr>
<tr><td colspan="6">Median age</td><td colspan="6">Median age</td><td></td></tr>
<tr><td colspan="6">47.0 (18.0) years</td><td colspan="6">47.0 (12.7) years</td><td>0.73</td></tr>
<tr><td colspan="6">Mean farming experience</td><td colspan="6">Mean farming experience</td><td></td></tr>
<tr><td colspan="6">25.0 (15.0) years</td><td colspan="6">30.0 (25.0) years</td><td>0.93</td></tr>
<tr><td colspan="3">Full-time</td><td colspan="3">Part-time</td><td colspan="3">Full-time</td><td colspan="3">Part-time</td><td></td></tr>
<tr><td colspan="3">96</td><td colspan="3">13</td><td colspan="3">9</td><td colspan="3">1</td><td>0.86</td></tr>
<tr><td colspan="6">Daily time spent at the farm</td><td colspan="6">Daily time spent at the farm</td><td></td></tr>
<tr><td colspan="6">15.0 (5.0) hours</td><td colspan="6">15.0 (5.2) hours</td><td>0.46</td></tr>
<tr><td colspan="2">Primary education</td><td colspan="2">Secondary and vocational education</td><td colspan="2">Tertiary education</td><td colspan="2">Primary education</td><td colspan="2">Secondary and vocational education</td><td colspan="2">Tertiary education</td><td></td></tr>
<tr><td colspan="2">19</td><td colspan="2">82</td><td colspan="2">8</td><td colspan="2">1</td><td colspan="2">7</td><td colspan="2">2</td><td>0.35</td></tr>
<tr><td colspan="3">Farming family tradition</td><td colspan="3">No farming family tradition</td><td colspan="3">Farming family tradition</td><td colspan="3">No farming family tradition</td><td></td></tr>
<tr><td colspan="3">95</td><td colspan="3">14</td><td colspan="3">9</td><td colspan="3">1</td><td>0.80</td></tr>
<tr><td colspan="3">Farm worker employment</td><td colspan="3">No farm worker employment</td><td colspan="3">Farm worker employment</td><td colspan="3">No farm worker employment</td><td></td></tr>
<tr><td colspan="3">31</td><td colspan="3">78</td><td colspan="3">3</td><td colspan="3">7</td><td>0.92</td></tr>
</table>

Table S6. Association of availability of ditch at the main entrance in 119 goat farms in Greece with socio-demographic characteristics of the farmers.

**Farms with no ditch at the main entrance (*n* = 119)**			**Farms with ditch at the main entrance (*n* = 0)**			***p***
Male	Female		Male	Female		
108	11		-	-		n/r
Median age			Median age			
46.0 (17.5) years			-			n/r
Mean farming experience			Mean farming experience			
25.0 (15.0) years			-			n/r
Full-time	Part-time		Full-time	Part-time		
105	14		-	-		n/r
Daily time spent at the farm			Daily time spent at the farm			
15.0 (5.0) hours			-			n/r
Primary education	Secondary and vocational education	Tertiary education	Primary education	Secondary and vocational education	Tertiary education	
20	89	10	-	-	-	n/r
Farming family tradition	No farming family tradition		Farming family tradition	No farming family tradition		
104	15		-	-		n/r
Farm worker employment	No farm worker employment		Farm worker employment	No farm worker employment		
34	85		-	-		n/r

Table S7. Association of maintenance of quarantine period for new animals into the farm in 119 goat farms in Greece with socio-demographic characteristics of the farmers.

**Farms with no maintenance of quarantine period for new animals (n = 58)**			**Farms with maintenance of quarantine period for new animals (n = 61)**			p
Male	Female		Male	Female		
55	3		53	8		0.13
Median age			Median age			
48.0 (16.7) years			46.0 (21.0 years)			0.33
Mean farming experience			Mean farming experience			
30.0 (13.8) years			20.0 (30.0) years			0.0002
Full-time	Part-time		Full-time	Part-time		
52	6		53	8		0.64
Daily time spent at the farm			Daily time spent at the farm			
15.0 (5.0) hours			15.0 (5.0) hours			0.63
Primary education	Secondary and vocational education	Tertiary education	Primary education	Secondary and vocational education	Tertiary education	
9	46	3	11	43	7	0.40
Farming family tradition	No farming family tradition		Farming family tradition	No farming family tradition		
54	4		50	11		0.07
Farm worker employment	No farm worker employment		Farm worker employment	No farm worker employment		
8	50		26	35		0.0005

Table S8. Association of maintenance of isolation for sick animals in 119 goat farms in Greece with socio-demographic characteristics of the farmers.

<table>
<tr><th colspan="6">Farms with no maintenance of isolation for sick animals (n = 21)</th><th colspan="6">Farms with maintenance of isolation for sick animals (n = 98)</th><th>p</th></tr>
<tr><td colspan="3">Male</td><td colspan="3">Female</td><td colspan="3">Male</td><td colspan="3">Female</td><td></td></tr>
<tr><td colspan="3">20</td><td colspan="3">1</td><td colspan="3">88</td><td colspan="3">10</td><td>0.43</td></tr>
<tr><td colspan="6">Median age</td><td colspan="6">Median age</td><td></td></tr>
<tr><td colspan="6">47.0 (17.0) years</td><td colspan="6">46.0 (17.7) years</td><td>0.94</td></tr>
<tr><td colspan="6">Mean farming experience</td><td colspan="6">Mean farming experience</td><td></td></tr>
<tr><td colspan="6">30.0 (5.0) years</td><td colspan="6">25.0 (18.7) years</td><td>0.21</td></tr>
<tr><td colspan="3">Full-time</td><td colspan="3">Part-time</td><td colspan="3">Full-time</td><td colspan="3">Part-time</td><td></td></tr>
<tr><td colspan="3">19</td><td colspan="3">2</td><td colspan="3">86</td><td colspan="3">12</td><td>0.73</td></tr>
<tr><td colspan="6">Daily time spent at the farm</td><td colspan="6">Daily time spent at the farm</td><td></td></tr>
<tr><td colspan="6">15.0 (7.0) hours</td><td colspan="6">15.0 (5.0) hours</td><td>0.70</td></tr>
<tr><td colspan="2">Primary education</td><td colspan="2">Secondary and vocational education</td><td colspan="2">Tertiary education</td><td colspan="2">Primary education</td><td colspan="2">Secondary and vocational education</td><td colspan="2">Tertiary education</td><td></td></tr>
<tr><td colspan="2">4</td><td colspan="2">15</td><td colspan="2">2</td><td colspan="2">16</td><td colspan="2">74</td><td colspan="2">8</td><td>0.93</td></tr>
<tr><td colspan="3">Farming family tradition</td><td colspan="3">No farming family tradition</td><td colspan="3">Farming family tradition</td><td colspan="3">No farming family tradition</td><td></td></tr>
<tr><td colspan="3">18</td><td colspan="3">3</td><td colspan="3">86</td><td colspan="3">12</td><td>0.80</td></tr>
<tr><td colspan="3">Farm worker employment</td><td colspan="3">No farm worker employment</td><td colspan="3">Farm worker employment</td><td colspan="3">No farm worker employment</td><td></td></tr>
<tr><td colspan="3">1</td><td colspan="3">20</td><td colspan="3">33</td><td colspan="3">65</td><td>0.008</td></tr>
</table>

Table S9. Association of carcass disposal according to relevant regulations in 119 goat farms in Greece with socio-demographic characteristics of the farmers.

<table>
<tr><th colspan="6">Farms with no carcass disposal according to relevant regulations (n = 69)</th><th colspan="6">Farms with carcass disposal according to relevant regulations (n = 50)</th><th>p</th></tr>
<tr><td colspan="3">Male</td><td colspan="3">Female</td><td colspan="3">Male</td><td colspan="3">Female</td><td></td></tr>
<tr><td colspan="3">64</td><td colspan="3">5</td><td colspan="3">44</td><td colspan="3">6</td><td>0.38</td></tr>
<tr><td colspan="6">Median age</td><td colspan="6">Median age</td><td></td></tr>
<tr><td colspan="6">45 (18.0) years</td><td colspan="6">47.0 (16.5) years</td><td>0.66</td></tr>
<tr><td colspan="6">Mean farming experience</td><td colspan="6">Mean farming experience</td><td></td></tr>
<tr><td colspan="6">25.0 (10.0) years</td><td colspan="6">25.0 (35.0) years</td><td>0.53</td></tr>
<tr><td colspan="3">Full-time</td><td colspan="3">Part-time</td><td colspan="3">Full-time</td><td colspan="3">Part-time</td><td></td></tr>
<tr><td colspan="3">63</td><td colspan="3">6</td><td colspan="3">42</td><td colspan="3">8</td><td>0.22</td></tr>
<tr><td colspan="6">Daily time spent at the farm</td><td colspan="6">Daily time spent at the farm</td><td></td></tr>
<tr><td colspan="6">15.0 (5.0) hours</td><td colspan="6">15.0 (5.0) hours</td><td>0.53</td></tr>
<tr><td colspan="2">Primary education</td><td colspan="2">Secondary and vocational education</td><td colspan="2">Tertiary education</td><td colspan="2">Primary education</td><td colspan="2">Secondary and vocational education</td><td colspan="2">Tertiary education</td><td></td></tr>
<tr><td colspan="2">12</td><td colspan="2">49</td><td colspan="2">8</td><td colspan="2">8</td><td colspan="2">40</td><td colspan="2">2</td><td>0.31</td></tr>
<tr><td colspan="3">Farming family tradition</td><td colspan="3">No farming family tradition</td><td colspan="3">Farming family tradition</td><td colspan="3">No farming family tradition</td><td></td></tr>
<tr><td colspan="3">61</td><td colspan="3">8</td><td colspan="3">43</td><td colspan="3">7</td><td>0.70</td></tr>
<tr><td colspan="3">Farm worker employment</td><td colspan="3">No farm worker employment</td><td colspan="3">Farm worker employment</td><td colspan="3">No farm worker employment</td><td></td></tr>
<tr><td colspan="3">20</td><td colspan="3">49</td><td colspan="3">14</td><td colspan="3">36</td><td>0.91</td></tr>
</table>

Table S10. Association of application of rodenticides in 119 goat farms in Greece with socio-demographic characteristics of the farmers.

**Farms with no application of rodenticides (n = 69)**			**Farms with application of rodenticides (n = 50)**			p
Male	Female		Male	Female		
63	6		45	5		0.81
Median age			Median age			
46.0 (18.0) years			46.5 (17.0) years			0.99
Mean farming experience			Mean farming experience			
25.0 (25.0) years			30.0 (25.0) years			0.43
Full-time	Part-time		Full-time	Part-time		
60	9		45	5		0.61
Daily time spent at the farm			Daily time spent at the farm			
15.0 (6.0) hours			15.0 (5.0) hours			0.48
Primary education	Secondary and vocational education	Tertiary education	Primary education	Secondary and vocational education	Tertiary education	
14	49	6	6	40	4	0.47
Farming family tradition	No farming family tradition		Farming family tradition	No farming family tradition		
61	8		43	7		0.70
Farm worker employment	No farm worker employment		Farm worker employment	No farm worker employment		
22	47		38	12		0.35

Table S11. Association of application of systemic disinfections in 119 goat farms in Greece with socio-demographic characteristics of the farmers.

<table>
<tr><th colspan="6">Farms with no application of systemic disinfections (n = 17)</th><th colspan="6">Farms with application of systemic disinfections (n = 102)</th><th>p</th></tr>
<tr><td colspan="3">Male</td><td colspan="3">Female</td><td colspan="3">Male</td><td colspan="3">Female</td><td></td></tr>
<tr><td colspan="3">16</td><td colspan="3">1</td><td colspan="3">92</td><td colspan="3">10</td><td>0.61</td></tr>
<tr><td colspan="6">Median age</td><td colspan="6">Median age</td><td></td></tr>
<tr><td colspan="6">52.0 (15.0) years</td><td colspan="6">46.0 (18.0) years</td><td>0.46</td></tr>
<tr><td colspan="6">Mean farming experience</td><td colspan="6">Mean farming experience</td><td></td></tr>
<tr><td colspan="6">30.0 (10.0) years</td><td colspan="6">25.0 (15.0) years</td><td>0.75</td></tr>
<tr><td colspan="3">Full-time</td><td colspan="3">Part-time</td><td colspan="3">Full-time</td><td colspan="3">Part-time</td><td></td></tr>
<tr><td colspan="3">17</td><td colspan="3">0</td><td colspan="3">88</td><td colspan="3">14</td><td>0.10</td></tr>
<tr><td colspan="6">Daily time spent at the farm</td><td colspan="6">Daily time spent at the farm</td><td></td></tr>
<tr><td colspan="6">15.0 (5.0) hours</td><td colspan="6">15.0 (5.0) hours</td><td>0.91</td></tr>
<tr><td colspan="2">Primary education</td><td colspan="2">Secondary and vocational education</td><td colspan="2">Tertiary education</td><td colspan="2">Primary education</td><td colspan="2">Secondary and vocational education</td><td colspan="2">Tertiary education</td><td></td></tr>
<tr><td colspan="2">7</td><td colspan="2">7</td><td colspan="2">3</td><td colspan="2">13</td><td colspan="2">82</td><td colspan="2">7</td><td>0.002</td></tr>
<tr><td colspan="3">Farming family tradition</td><td colspan="3">No farming family tradition</td><td colspan="3">Farming family tradition</td><td colspan="3">No farming family tradition</td><td></td></tr>
<tr><td colspan="3">16</td><td colspan="3">1</td><td colspan="3">88</td><td colspan="3">14</td><td>0.37</td></tr>
<tr><td colspan="3">Farm worker employment</td><td colspan="3">No farm worker employment</td><td colspan="3">Farm worker employment</td><td colspan="3">No farm worker employment</td><td></td></tr>
<tr><td colspan="3">1</td><td colspan="3">16</td><td colspan="3">33</td><td colspan="3">69</td><td>0.025</td></tr>
</table>

Table S12. Association of grazing practice grazing in 119 goat farms in Greece with socio-demographic characteristics of the farmers.

**Farms with grazing practice (*n* = 112)**			**Farms with no grazing practice (*n* = 7)**			***p***
Male	Female		Male	Female		
103	9		5	2		0.07
Median age			Median age			
47.0 (17.0) years			42.0 (17.0) years			0.19
Mean farming experience			Mean farming experience			
26.0 (15.0) years			0.0 (10.0) years			0.005
Full-time	Part-time		Full-time	Part-time		
98	14		7	0		0.32
Daily time spent at the farm			Daily time spent at the farm			
15.0 (5.0) hours			8.0 (3.0) hours			0.010
Primary education	Secondary and vocational education	Tertiary education	Primary education	Secondary and vocational education	Tertiary education	
19	83	10	1	6	0	0.68
Farming family tradition	No farming family tradition		Farming family tradition	No farming family tradition		
99	13		5	2		0.19
Farm worker employment	No farm worker employment		Farm worker employment	No farm worker employment		
28	84		6	1		0.0006

Table S13. Association of common grazing of farm animals with wildlife ruminants in 119 goat farms in Greece with socio-demographic characteristics of the farmers.

**Farms with common grazing of farm animals with wildlife ruminants (n = 11)**			**Farms with no common grazing of farm animals with wildlife ruminants (n = 108)**			p
Male	Female		Male	Female		
11	0		97	11		0.27
Median age			Median age			
43.0 (17.0) years			46.5 (17.2) years			0.76
Mean farming experience			Mean farming experience			
30.0 (5.0) years			25.0 (16.2) years			0.41
Full-time	Part-time		Full-time	Part-time		
8	3		97	11		0.09
Daily time spent at the farm			Daily time spent at the farm			
15.0 (0.0) hours			15.0 (6.2) hours			0.10
Primary education	Secondary and vocational education	Tertiary education	Primary education	Secondary and vocational education	Tertiary education	
3	7	1	17	82	9	0.61
Farming family tradition	No farming family tradition		Farming family tradition	No farming family tradition		
9	2		94	14		0.63
Farm worker employment	No farm worker employment		Farm worker employment	No farm worker employment		
3	8		31	77		0.92

Table S14. Association of presence of wild carnivore mammals near 119 goat farms in Greece with socio-demographic characteristics of the farmers.

**Farms with presence of wild carnivore animals near the farm (*n* = 98)**			**Farms with no presence of wild carnivore animals near the farm (*n* = 21)**			***p***
Male	Female		Male	Female		
93	5		15	6		0.0008
Median age			Median age			
47.0 (17.7) years			45.0 (17.0) years			0.99
Mean farming experience			Mean farming experience			
26.0 (15.0) years			25.0 (25.0) years			0.68
Full-time	Part-time		Full-time	Part-time		
84	14		21	0		0.07
Daily time spent at the farm			Daily time spent at the farm			
15.0 (5.0) hours			12.0 (8.0) hours			0.15
Primary education	Secondary and vocational education	Tertiary education	Primary education	Secondary and vocational education	Tertiary education	
17	73	8	3	16	2	0.93
Farming family tradition	No farming family tradition		Farming family tradition	No farming family tradition		
85	13		18	3		0.90
Farm worker employment	No farm worker employment		Farm worker employment	No farm worker employment		
27	71		7	14		0.59

Table S15. Association of presence of hunters in the area around the farm in 119 goat farms in Greece with socio-demographic characteristics of the farmers.

**Farms with presence of hunters in the area around (*n* = 113)**			**Farms with no presence of hunters in the area around (*n* = 6)**			***p***
Male	Female		Male	Female		
102	11		6	0		0.42
Median age			Median age			
46.0 (17.0) years			60 (13.2) years			0.11
Mean farming experience			Mean farming experience			
25.0 (15.0) years			35 (23.8) years			0.11
Full-time	Part-time		Full-time	Part-time		
101	12		4	2		0.09
Daily time spent at the farm			Daily time spent at the farm			
15.0 (6.0) hours			15 (3.7) hours			0.31
Primary education	Secondary and vocational education	Tertiary education	Primary education	Secondary and vocational education	Tertiary education	
20	85	8	0	4	2	0.06
Farming family tradition	No farming family tradition		Farming family tradition	No farming family tradition		
98	15		6	0		0.34
Farm worker employment	No farm worker employment		Farm worker employment	No farm worker employment		
31	82		3	3		0.23

Table S16. Association of transhumance in 119 goat farms in Greece with socio-demographic characteristics of the farmers.

**Farms with transhumance (*n* = 28)**			**Farms with no transhumance (*n* = 91)**			***p***
Male	Female		Male	Female		
28	0		80	11		0.05
Median age			Median age			
48.0 (18.8) years			46.0 (17.0) years			0.63
Mean farming experience			Mean farming experience			
30 (16.2) years			46.0 (17.0) years			0.028
Full-time	Part-time		Full-time	Part-time		
25	3		80	11		0.84
Daily time spent at the farm			Daily time spent at the farm			
15.0 (0.8) hours			15.0 (6.5) hours			0.015
Primary education	Secondary and vocational education	Tertiary education	Primary education	Secondary and vocational education	Tertiary education	
8	17	3	12	72	7	0.12
Farming family tradition	No farming family tradition		Farming family tradition	No farming family tradition		
28	0		76	15		0.002
Farm worker employment	No farm worker employment		Farm worker employment	No farm worker employment		
7	21		27	64		0.63

Table S17. Association of presence of spots suitable for vector′ reproduction and development in 119 goat farms in Greece with socio-demographic characteristics of the farmers.

**Farms with presence of spots suitable for vector′ reproduction and development (*n* = 83)**			**Farms with no presence of spots suitable for vector′ reproduction and development (*n* = 36)**			***p***
Male	Female		Male	Female		
74	9		34	2		0.36
Median age			Median age			
46.0 (18.0) years			47.0 (16.3) years			0.69
Mean farming experience			Mean farming experience			
25.0 (15.0) years			25.0 (21.3) years			0.98
Full-time	Part-time		Full-time	Part-time		
71	12		34	2		0.17
Daily time spent at the farm			Daily time spent at the farm			
15.0 (7.0) hours			15.0 (5.0) hours			0.46
Primary education	Secondary and vocational education	Tertiary education	Primary education	Secondary and vocational education	Tertiary education	
14	62	7	6	27	3	0.99
Farming family tradition	No farming family tradition		Farming family tradition	No farming family tradition		
74	9		30	6		0.38
Farm worker employment	No farm worker employment		Farm worker employment	No farm worker employment		
23	60		11	25		0.81

Table S18. Association of purchase of replacement animals in 119 goat farms in Greece with socio-demographic characteristics of the farmers.

**Farms with purchase of replacement animals (*n* = 41)**			**Farms with no purchase of replacement animals (*n* = 78)**			***p***
Male	Female		Male	Female		
37	4		71	7		0.89
Median age			Median age			
45.0 (18.0) years			48.0 (17.0) years			0.38
Mean farming experience			Mean farming experience			
20.0 (30.0) years			30.0 (20.0) years			0.005
Full-time	Part-time		Full-time	Part-time		
36	5		69	9		0.92
Daily time spent at the farm			Daily time spent at the farm			
15.0 (5.0) hours			15.0 (5.0) hours			0.97
Primary education	Secondary and vocational education	Tertiary education	Primary education	Secondary and vocational education	Tertiary education	
10	26	5	10	63	5	0.12
Farming family tradition	No farming family tradition		Farming family tradition	No farming family tradition		
32	9		71	7		0.049
Farm worker employment	No farm worker employment		Farm worker employment	No farm worker employment		
20	21		14	64		0.0004

Table S19. Association of the overall score for biosecurity-related practices in 119 goat farms in Greece with socio-demographic characteristics of the farmers.

Socio-demographic characteristics of farmers	r_{sp}	p
Farmer gender	0.256	0.005
Farmer age	0.036	0.70
Length of farming experience	–0.126	0.17
Professional involvement in farming	–0.073	0.43
Daily period of presence at the farm	–0.088	0.34
Farmer education level	0.100	0.28
Family farming-tradition	0.118	> 0.20
Presence of working staff at the farm	–0.219	0.017
